# Mitigation potential of urban greening during heatwaves and stormwater events: a modeling study for Karlsruhe, Germany

**DOI:** 10.1038/s41598-025-89842-z

**Published:** 2025-02-13

**Authors:** Rocco Pace, Theodore A. Endreny, Marco Ciolfi, Marcel Gangwisch, Somidh Saha, Nadine K. Ruehr, Rüdiger Grote

**Affiliations:** 1 Institute of Meteorology and Climate Research, Atmospheric Environmental Research (IMK-IFU), Karlsruhe Institute of Technology (KIT), Garmisch-Partenkirchen, Germany; 2 EURAC Research, Institute for Renewable Energy, Bozen-Bolzano, Italy; 3 Department of Environmental Resources Engineering, SUNY ESF, Syracuse, NY USA; 4 Institute of Research on Terrestrial Ecosystems (IRET), National Research Council (CNR), Porano, Italy; 5 Research Centre Human Biometeorology, German Meteorological Service (DWD), Freiburg, Germany; 6 Institute of Earth and Environmental Sciences, Faculty of Environment and Natural Resources, University of Freiburg, Freiburg, Germany; 7 Institute of Geography and Geoecology (IfGG), Karlsruhe Institute of Technology (KIT), Karlsruhe, Germany; 8 Institute for Technology Assessment and Systems Analysis (ITAS), Karlsruhe Institute of Technology, Karlsruhe, Germany

**Keywords:** Ecosystem services, Ecological modelling, Urban ecology

## Abstract

Climate change is increasing the frequency and intensity of urban heat islands and stormwater flooding. In order to mitigate these threats cities are turning toward green infrastructure to restore the hydrologic cycle in a way that increases the ecosystem services provided by trees. Strategically designed green infrastructure can mitigate runoff volume by rainfall interception through tree canopies and redirect impervious runoff into bioswales that promote infiltration. In addition, urban greens mitigate extreme heat via evapotranspiration and shading. Here we applied the i-Tree HydroPlus model to the German city of Karlsruhe and its twenty-seven districts with varying initial conditions of tree cover to analyze the potential for both runoff and heat mitigation during dry and wet periods throughout a 5-year period. After analyzing initial tree cover and drainage conditions, we used the model to simulate a green infrastructure scenario for each district with restored hydrology and tree cover at 30%. Regarding trade-offs between runoff and heat mitigation, the results confirm that dry soils before storm events lead to greater runoff reduction by 10%, and wet soils prior to heatwaves resulted in a greater evaporative cooling. Compared to current conditions, the green infrastructure scenarios resulted in decreasing the number of extreme heat hours (Heat Index > 31 °C) per year on average by 64.5%, and to reduce runoff in average by 58% across all city districts. Thus, our simulation results show that investing into a greener infrastructure, has positive impacts on microclimate and hydrology. Finally, we discuss synergies and trade-offs of the investigated management options as well as the transferability of results to other cities.

## Introduction

Climate change not only leads to increased average temperatures, but also an increased frequency and intensity of extreme events such as heatwaves, droughts, and flash floods^1,2^. Cities are particularly exposed to these effects, due to large amounts of impervious surfaces that retain heat and channel water out of the system, small coverage with vegetation and water, and human activities that generate heat^3^. Cities also contain large numbers of people, and their health and wellbeing are jeopardized by extreme heat^4–6^, including increased vulnerability to respiratory disease from urban air pollution^7,8^. The extreme heat and drought stress may also provoke a shift in species composition of urban plant communities^9,10^ and lead to greater biogenic emissions which contribute to air pollution^11^. The spatially extensive and connected impervious cover across cities prevents rainfall from infiltrating into soils and replaces natural drainage networks with storm drains and sewers that reduce the availability for evapotranspiration and its associated cooling effect^12^. During extreme precipitation events, these artificial drainage networks often experience severe flooding from stormwater runoff^13,14^.

In order to reduce the vulnerability of urban areas to extreme heat, drought, and heavy rainfall, it has been suggested to increase the abundance of green infrastructure^12,15^ and to decrease the degree of connected impervious cover^16^. Properly designed, green infrastructure has the potential to restore the hydrological cycle by directing runoff from impervious areas to soil-vegetative systems such as bioswales or rain gardens that capture, pond, infiltrate, and evapotranspire the stormwater^17^. In this respect, green infrastructure may represent a sustainable drainage system, where trees and the soil below them will capture and use stormwater runoff, e.g. to cool the air via evapotranspiration, a process that converts environmental heat into water vapor provided that sufficient soil water is available^12^. Tree canopy that extends over impervious surfaces also directly leads to cooler surfaces by shielding them from direct solar radiation, and the cooler surfaces then emit less heat into the surrounding air^18^. Tree canopies can intercept rainfall, while throughfall onto pervious soil rather than impervious areas will contribute to more infiltration, and lead to a reduction in stormwater runoff^19,20^. Therefore, urban greening that leads to increased tree cover, decreased impervious cover, and a restored hydrological cycle will contribute to the ecosystem services of heat- and runoff reduction, two processes that are often investigated separately yet are, in fact, inherently related^21^. Feedback between them includes, for example, that trees and the pervious ground area around them allow for additional storage of rainfall in the soil and in the plant canopy preventing flooding^22^, while on the other hand the stored water enables trees to provide direct and indirect cooling by enabling transpiration and preventing leaf shedding^23^. It is, therefore, sensible to investigate the impact of urban greening on both ecosystem services together.

Soil moisture content is an important boundary condition that regulates the effectiveness of urban trees in reducing air temperature and urban soils in reducing stormwater runoff. The evapotranspiration process of trees depends on root zone soil moisture levels, which are above what is called the wilting point^24,25^. Accordingly, models with the objective to evaluate the heat mitigation effects of urban vegetation need to consider the degree of soil water depletion^23^. In contrast, a water-depleted soil, and a permeable area with a deeper groundwater table, has in principle a higher ability to infiltrate and store rainfall and stormwater and thus prevent runoff. Infiltration will not occur in groundwater-saturated soils or when rainfall rates exceed the soil infiltration rate, which is a function of soil physical properties (e.g., hydraulic conductivity)^26^.

To reduce incidents of extreme heat and flooding many cities plan to naturalize the hydrological cycle and increase the number of trees and the area covered by plant canopies^27^ using green infrastructure-type designs, also called urban greening or nature-based solutions. One tree cover target widely promoted is a minimum of 30% tree coverage in each developed or neighborhood area containing urban residents^28^, which is a baseline target to deliver ecosystem services that benefit human health^29^. Unfortunately, most cities globally are losing canopy and pervious area rather than seeing an increase associated with urban greening^30^. Despite global trends in the loss of urban tree cover and the increase in extreme weather, local climatic and hydrological conditions need to be assessed to quantify the benefits of tree planting and explore the feasibility of tree viability within green infrastructure projects. An excellent example of this need for local analysis was the Green Lungs or *GrüneLunge* inter- and transdisciplinary project (https://www.projekt-gruenelunge.de/english/index.php) in the City of Karlsruhe in Southern Germany. The city strives to decrease its vulnerability against expected climate extremes and increase the benefits from urban and peri-urban forests^31^. Various ecosystem services about trees and forests have already been highlighted^32^, but knowledge about quantitative cause-effect relationships and eventual trade-offs is still missing.

The study aims is to develop a framework for examining the interrelated ecosystem services of soil moisture in mitigating runoff and extreme heat across a range of climatological and land cover scenarios. The experiences gained should serve to illustrate analysis and management options for most cities. Specifically, we will use a water and energy balance city-scale hydro-climatology model to assess to which degree green infrastructure can reduce extreme heat (i.e., air temperature and humidity during the warm season) and stormwater runoff. The model i-Tree HydroPlus, which consists of a hydrological (i-Tree Hydro) and a climate part (i-Tree Cool Air), has been first applied to case studies in the United States for runoff^19,33^ or heat mitigation assessments^34–36^. While either using the climate part to investigate the role of vegetation for temperature development^37–39^, or using the hydrology part for evaluating the impact on runoff the model has been widely applied in Europe, South America, and China^40–42^. However, it has never been used for a joint evaluation of both impacts simultaneously. In the current contribution, we particular emphasize on the influence of boundary conditions on the ecosystem services of green infrastructure, in particular on the effect of soil moisture and the percentage of green cover. Since heat and drought often occur together^43,44^ and should increase in intensity and frequency with climate change, depicting possible synergies or trade-offs that may affect the degree of ecosystem service benefit with different management options is highly relevant. Our hypotheses are that (1) dry conditions will decrease the heat mitigation effect provided by trees during very warm periods but improve flood protection due to enhanced infiltration, and (2) increasing tree canopy and pervious cover decreases the incidences of extreme heat and stormwater runoff. To test these hypotheses and explore the extreme heat and stormwater runoff changes associated with land cover, we applied a numerical simulation model i-Tree HydroPlus under current climate conditions using either the current vegetation cover or one based on an urban greening scenario.

## Methods

### Study area and workflow

**Fig. 1:**
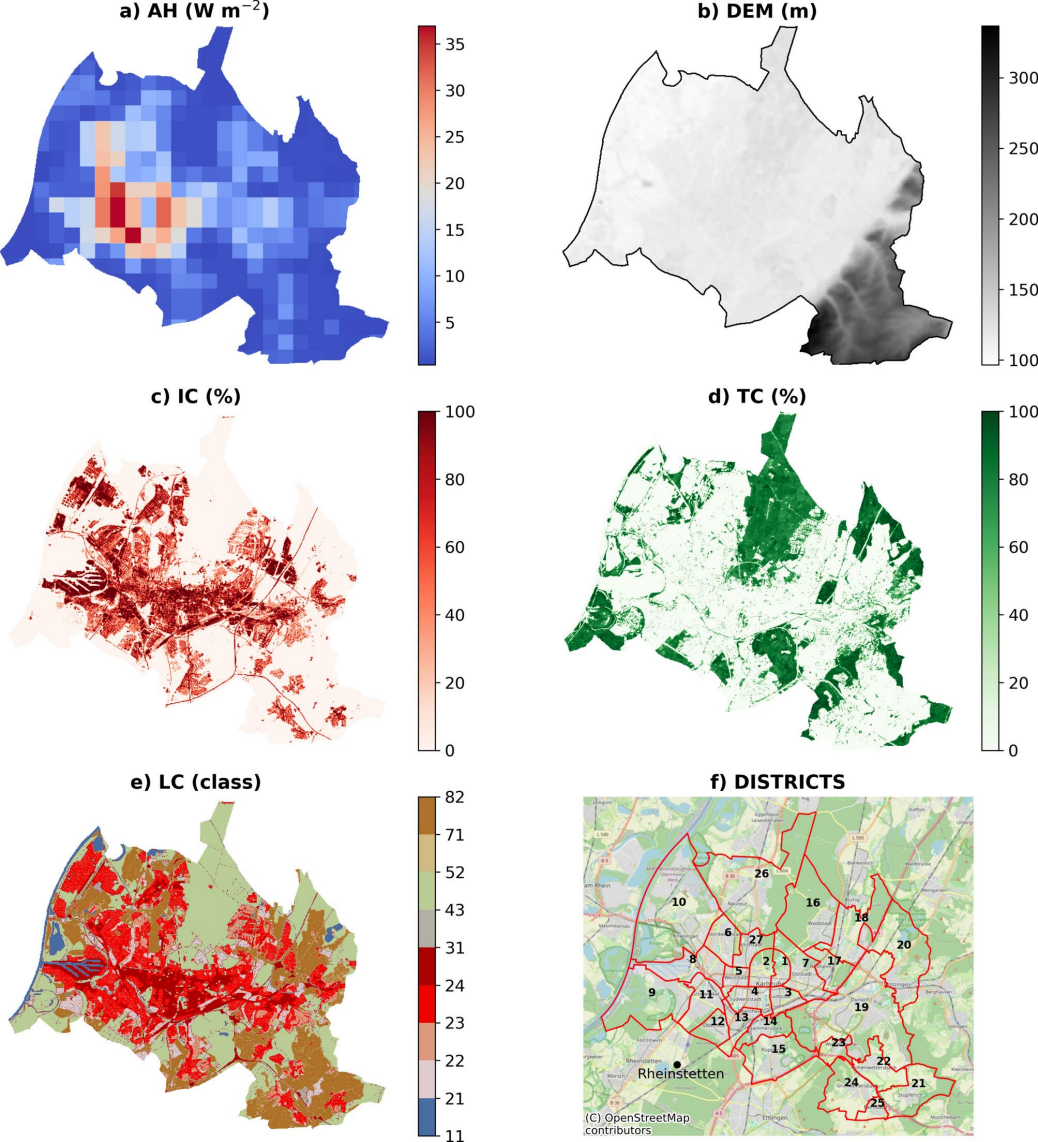
Spatial input data for the i-Tree HydroPlus models included: (**a**) Anthropogenic Heat (AH), (**b**) Digital Elevation Model (DEM), (**c**) Impervious Cover (IC), (**d**) Tree Cover (TC), (**e**) US NLCD Land Cover Classes (LC), and (**f**) the Map of Karlsruhe Districts (see Table 1 for identification numbers). The maps were generated using Python 3.11.4 (https://www.python.org).

The city of Karlsruhe is situated in the southwest of Germany within the Upper Rhine Valley. The urban area extends for 173.4 km^2^ with a residential population of about 304,000 inhabitants^45^. The municipality is divided into 27 districts (Table 1), with different population densities (particularly high in the Durlach district and the inner city), and different age class distribution of the people (with the oldest classes > 65 years mostly in rural areas (see S1)). The average elevation is 115 m above sea level, with relatively flat terrain over most of the area, except in the southeast where the elevation increases significantly (Fig. 1b). The climatic category is Cfb according to the Köppen-Geiger classification, indicating a warm temperate climate (C) characterized by high humidity levels (f) and warm summers (b)^46^. The main land cover classes, based on the reclassification from the Urban Atlas 2018 (UA) into the National Land Cover Database (NLCD) classification (see Pace et al.^37^) (Figs. 1e, S2), are developed areas (42.8%) that can be found predominantly in the city center. More forested areas that cover 32.7% of the city are mainly concentrated in the Waldstadt district in the north, while crops and agricultural fields (18.9%) are located in the surroundings.

**Table 1:** Land cover statistics for the German city of Karlsruhe districts relative to the total area, and only considering developed classes (21–24) [IC = impervious cover; TC = tree cover].

N	Name	Total area							Developed land cover classes		
		1° Land cover	Area	Area	Population	Pop. > = 65 yr	Mean IC	Mean TC	Mean IC	Mean TC	Area
		Class	x km^2^	(%)	N°	N°	(%)	(%)	(%)	(%)	(ha)
1	Innenstadt-Ost	43	1.6	0.9	6480	793	41	26	57.9	8.1	107.55
2	Innenstadt-West	43	2.41	1.4	10,091	1151	43	29	73.8	4.3	130.77
3	Südstadt	24	2.14	1.2	20,204	2397	68	4	70.5	2.7	196.83
4	Südweststadt	24	2.98	1.7	21,136	3141	57	9	70.5	3.1	232.47
5	Weststadt	24	1.73	1.0	20,093	3093	62	5	66.2	4.4	157.41
6	Nordweststadt	23	3.51	2.0	11,676	2569	44	4	54.2	3.5	275.94
7	Oststadt	43	5.27	3.0	19,683	2451	41	22	59.1	4.6	347.49
8	Mühlburg	24	5.32	3.1	16,610	3098	59	5	73.7	2.0	409.14
9	Daxlanden	43	10.92	6.3	11,288	3068	15	42	41.0	9.8	400.32
10	Knielingen	23	20.63	11.9	11,537	2069	28	17	60.7	3.4	891
11	Grünwinkel	23	4.4	2.5	11,306	2412	53	10	68.6	2.0	343.17
12	Oberreut	43	2.43	1.4	10,023	2229	25	33	45.3	7.7	133.02
13	Beiertheim-Bulach	24	2.88	1.7	7111	1387	44	6	52.1	4.0	238.41
14	Weiherfeld-Dammerstock	43	3.05	1.8	5952	1652	20	45	42.8	7.2	137.88
15	Rüppurr	82	6.99	4.0	10,963	2679	20	24	40.2	6.9	336.06
16	Waldstadt	43	10.38	6.0	12,224	2943	8	65	35.8	17.7	210.6
17	Rintheim	23	3.35	1.9	6336	1237	32	23	49.7	3.8	202.77
18	Hagsfeld	43	7.29	4.2	7146	1302	24	34	54.3	3.8	300.42
19	Durlach	43	22.86	13.2	31,173	6766	19	31	44.9	11.1	927.99
20	Grötzingen	43	11.33	6.5	9122	2337	13	38	49.7	9.3	283.41
21	Stupferich	82	6.46	3.7	2981	714	9	24	42.0	5.1	128.43
22	Hohenwettersbach	82	4.12	2.4	3038	555	8	7	27.8	6.1	115.11
23	Wolfartsweier	82	1.88	1.1	3149	760	19	21	46.1	5.9	75.06
24	Grünwettersbach	82	6.26	3.6	4148	1042	10	30	43.6	8.7	144.36
25	Palmbach	82	1.36	0.8	1974	376	19	3	48.2	1.6	54.27
26	Neureut	43	19.22	11.1	19,188	4176	16	39	55.5	4.4	520.29
27	Nordstadt	43	2.66	1.5	9357	1458	29	25	45.1	6.9	162.72

Primary (1°) Land Cover class uses National Land Cover Database codes, and district numbers (N°) serve as reference for included maps.

The workflow comprises several steps that outline the adopted methodology, illustrated in S3 and detailed in the following paragraphs. First, two coupled hydro-climatology models (Models) were implemented to support water and energy balances under different scenarios. Next, spatial land cover and meteorological data were processed as inputs (Inputs) to support the scenarios. Then model outputs were compared with observed mobile air temperature data for validation (Evaluation with observed data). Subsequently, two extreme events—characterized by very hot and dry conditions, and heavy rainfall with wet conditions—were analyzed to test the hypothesis (Extreme events analysis). Finally, two key ecosystem services, the reduction of apparent temperature and avoided runoff, were assessed under different tree cover scenarios (Tree cover scenarios).

### Hydro-climatology models for water and energy balances

We used the hydro-climatology models called i-Tree Hydro^19^ and i-Tree Cool Air^34^ that are available within the i-Tree HydroPlus suite (https://www.itreetools.org/tools/research-suite) to evaluate the impacts of urban greening during extreme heat and rainfall events. i-Tree HydroPlus includes process-based environmental models that share common algorithms to track the water and energy balance during simulations of land cover changes, specifically considering vegetation processes. Model outputs include raster maps or time series of estimated air temperature, humidity, water runoff, as well as many storages and fluxes of water and energy such as soil moisture and latent heat.

The i-Tree Hydro model^19^, which is a statistically distributed urban water balance routine with various options for land cover characterization and runoff properties, specifically calculates sub-hourly to annual patterns in stormwater runoff throughout multiple years. The model covers the entire watershed considering general urban structure as well as orographic boundary conditions (such as slope). It uses tens of topographic index bins, each containing areas with hydrologically similar behavior, representing canopy interception, depression storage, infiltration, evapotranspiration, and runoff.

The i-Tree Cool Air model^34^ is a raster-based spatially distributed urban energy model that also considers the effects of water balance changes. Within each raster pixel, the energy balance solves for latent and sensible heat fluxes based on shortwave and longwave radiation, anthropogenic heat, and ground heat flux. Since runtime is inversely related to raster pixel resolution, we used a differentiated approach with three spatial resolutions for practical reasons: a single lumped area resolution with the i-Tree Hydro model representing 30 statistically indexed topographic indices (derived from 30 m resolution topography), and with the i-Tree Cool Air model a 30 m (for climate-specific event simulations), and a 300 m (for long-term simulations) horizontal raster resolution. The 300 m resolution raster was used for simulations with a duration of years when results would be spatially aggregated into the 27 district polygons, while the 30 m resolution raster was used for simulations with day to hour duration when results required more spatial detail.

### Model sensitivity, parameterization, and calibration

Most sensitive model parameters are related to properties of the pervious and the impervious cover, i.e. average depth to groundwater, surface depression storage, and directly connected impervious area (see Wang et al.^19^). Concerning the water balance, each of these terms is influencing how precipitation is partitioned into stormwater runoff or into infiltration and soil moisture reserves. For the energy balance, albedo and emissivity of land cover type and available liquid water determines how shortwave and longwave radiation were partitioned into the fluxes of ground heat, sensible heat, and latent heat.

Depression storage at the surface, which describes average stormwater ponding depth for pervious and impervious areas, is described by Rossman et al.^47^. We presumed that vegetation increases the storage due to litter accumulation and root effects on soil micro-topography, and assigned the following base case values from within known ranges^47^: pervious area with tree cover = 2.5 mm; pervious area with short vegetation = 2.0 mm; pervious area with only soil cover = 1.5 mm; impervious area with no vegetation = 0.5 mm; impervious area with tree cover = 1.5 mm. In addition to the base case, we established a green infrastructure case with sustainable urban drainage to bioswales where depression storage depths were increased to represent intentional efforts to pond water for infiltration. Here we set the surface storage at pervious areas with tree cover to 15 mm and pervious area with short vegetation to 12.5 mm.

The directly connected impervious area (DCIA), also known as the effective impervious area, represents the fraction of the total impervious area (TIA) that drains stormwater directly to the basin outlet via artificial pathways of roadside gutters or storm sewers. DCIA is calculated as DCIA = a(TIA)^b^ according to the method of Sutherland^48^, with DCIA and TIA given as relative values of the total simulated area, and coefficients a and b are assigned based on land cover conditions described in Rossman et al.^47^ and further elaborated in USEPA^49^. For the base case simulations with standard connections to sewers but residential rooftops not directly connected, we selected a = 0.1 and b = 1.5. For the green infrastructure case with sustainable urban drainage to bioswales, we represented highly disconnected basins with few storm sewer connections, setting a = 0.01 and b = 2.0. To clarify, in the green infrastructure scenario, even existing tree cover was retrofitted to function as bioswales. Based on an i-Tree Canopy (https://canopy.itreetools.org/) assessment of land cover in the study area, the total impervious area was approximately 31% of the total area, which yielded a DCIA fraction of 0.55 (or 17% of total area) for the base case and 0.31 (or 9.6% of total area) for the green infrastructure case. Runoff from disconnected impervious areas was sent to pervious areas.

The models were calibrated using two parameters such that they started and ended a 5-yr simulation with a 1.2 m average depth to groundwater (i.e., corresponding to the same average soil moisture deficit). Given how the models represent variation in groundwater depth across the domain using topographic index theory, this depth was selected to obtain an expression of surficial groundwater that approximated the river and lake area. The parameters of initial baseflow (i.e., Discharge_Subsurface_Initial_mph) and transmissivity at saturation (i.e., VadoseZone_Transmissivity_Max_m2ph) were auto-calibrated using PEST^50^, constraining the values to fit within the expected range based on local climatology data and topographic theory^51^. Different parameter values were obtained for the 30 m and 300 m resolution topographic data. Enforcing no change in groundwater depth is a standard hydrological assumption to simplify unknowns in annual or longer water balance analyses while allowing for variation in depth at each time step during the simulation.

Values for less sensitive model parameters were generally taken as default values as reported in Pace et al.^37^ and Yang et al.^34^. The parameters for soil physical properties were assigned based on Rawls et al.^52^ using a single soil texture for the study area, and then used by the model to implement the Green-Ampt infiltration routine^19^. Without a soil survey and knowing urban soil types are highly variable^53^, we selected a silty clay loam texture based on the study area having extensive floodplains with presumably a mix of sand, silt and clay alluvial soils. Based on Green-Ampt parameterization^52^ the silty clay loam was assigned a porosity of 0.47 cm^3^/cm^3^, saturation or effective porosity of 0.40 cm^3^/cm^3^, saturated hydraulic conductivity of 0.001 m/h, wetting front suction of 0.28 m, wilting point of 0.21 cm^3^/cm^3^, and field capacity of 0.34 cm^3^/cm^3^, which is the soil moisture content after gravitational drainage. This saturated hydraulic conductivity depends on texture class and is set to the lower end of those reported by Rawls et al.^52^ providing a conservative value for examining infiltration potential for stormwater. The model accounts for compaction through macropore fractions and evaporation depth but does not alter soil physical properties such as hydraulic conductivity or field capacity with changing soil moisture, consistent with findings by Pitt et al.^54^. Runoff partitioning is influenced by soil moisture through variable groundwater depth, and infiltration rates that decrease with soil moisture. Instantaneous runoff occurs either due to saturation excess, when the water table reaches the surface, or infiltration excess, when rainfall surpasses infiltration capacity. The soil rooting depth was set to 0.5 m for tree cover and 0.25 m for short vegetation, and for soils without vegetation evapotranspiration was effective to a soil depth of 0.2 m.

### Model inputs of map data and weather

Land cover data products of tree cover, impervious cover, and land cover class (e.g., urban, agriculture) were obtained from the European Space Agency Copernicus land cover data (CLMS) (https://land.copernicus.eu/). While other sources of such data were available, the CLMS were considered the best option for quality assurance and replication, due to their rigorous testing, widespread use among terrestrial scientists, and spatial continuity across all German cities.

The spatial domain considered by the model was the entire city of Karlsruhe, differentiated into 27 districts. The model was initialized with raster maps of a digital elevation model (m), tree canopy cover (%), impervious cover (%), and land cover classes using the Copernicus land cover database (Fig. 1). Specifically, the land cover classes (LC) are obtained from Urban Atlas (UA) (https://land.copernicus.eu/local/urban-atlas), tree cover (TC) and impervious cover (IC) from the High-Resolution Layers (https://land.copernicus.eu/en/products/high-resolution-layer-tree-cover-density, https://land.copernicus.eu/en/products/high-resolution-layer-imperviousness), and the Digital Elevation Model (DEM) from the digital surface model EU-DEM v1.0 (https://land.copernicus.eu/imagery-in-situ/eu-dem). Land cover classes from UA were converted into the NLCD classification^37^, and the spatial information was harmonized and resampled to a resolution of 30 m.

Anthropogenic heat flux was represented in sub-daily simulations using hourly values for the year 2010, resampled from the default 1-km resolution (Fig. 1a) provided by the AH4GUC dataset (https://urbanclimate.tse.ens.titech.ac.jp/ah4guc/)^55^.

District maps and statistics about the population were obtained from the department of the City of Karlsruhe (https://web6.karlsruhe.de/Stadtentwicklung/statistik/atlas/).

**Fig. 2:**
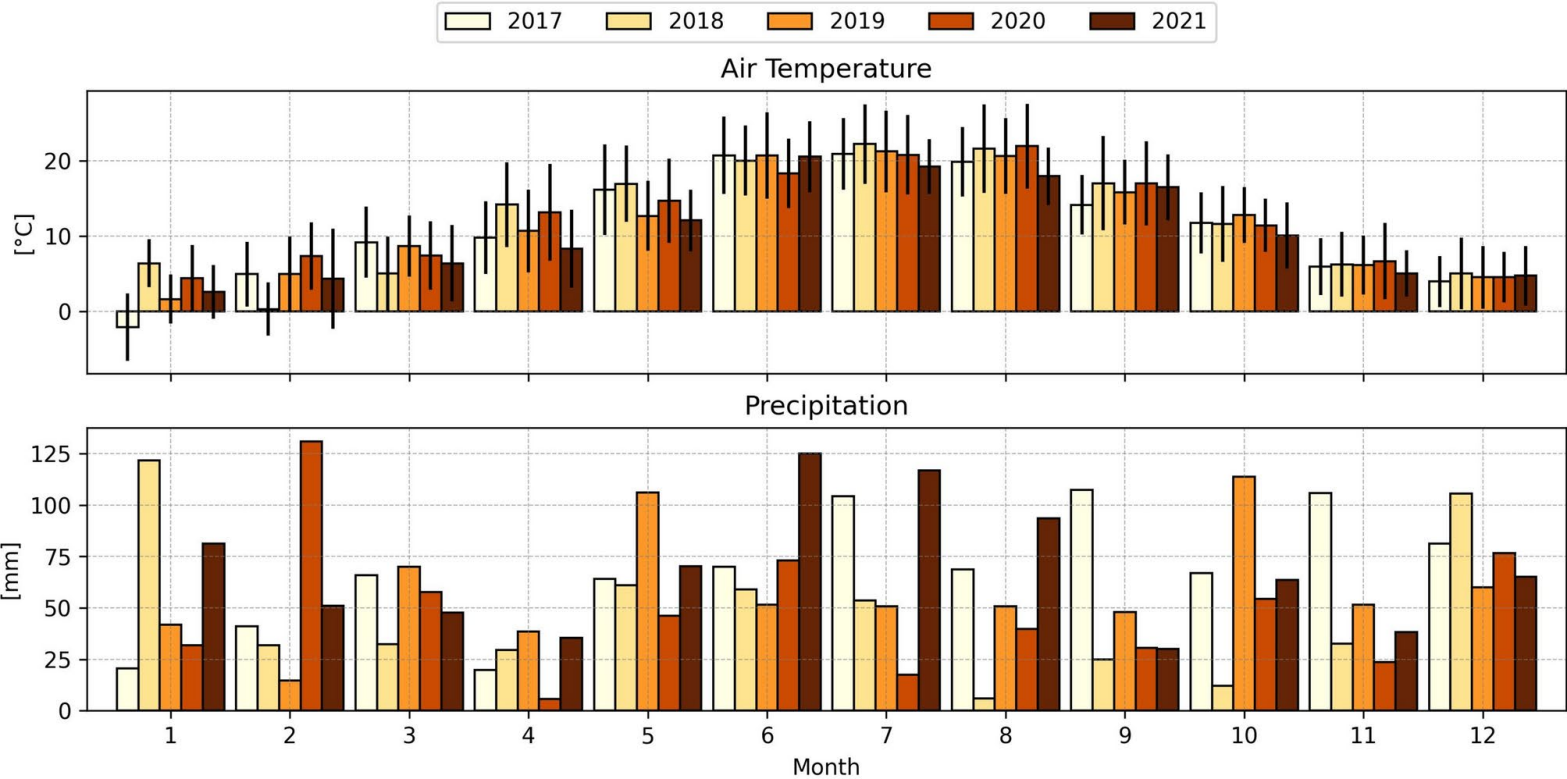
Climatology record for the study area of Karlsruhe, Germany with Top: monthly average air temperatures (2017–2021) with standard deviation lines, and Bottom: Monthly total precipitation (2017–2021).

Meteorological forcing data other than precipitation were recorded at the Karlsruhe-Baden-Baden airport USAF ID 107275 (see Fig. 2), downloaded from the National Centers for Environmental Information (NCEI) (ftp.ncei.noaa.gov/pub/data/noaa/), and processed using the i-Tree WeatherPrep utility^56^. Precipitation data were recorded by the close-by weather station at Rheinstetten (https://opendata.dwd.de/, Station-ID: 4177).

### Observational and evaluation data

Observed air temperature came from a measurement campaign reported by Gangwisch et al. (2023)^57^, which involved driving throughout representative areas of Karlsruhe on a 19.6 km route with a specially equipped vehicle (S4). Air temperature measurements at 2.0 m height above ground were conducted on June 25th 2020, between 00:00 and 12:00 (CEST), and on June 23rd 2020, between 12:00 and 24:00 (CEST). Data were recorded with a rate of 1 observation per second, and travel time averaged 20 min while maintaining a constant speed of 30 km/h in urban areas and 50 km/h in rural areas for highways. Measurements were preprocessed to correct possible spatial or temporal inaccuracies (e.g. GNSS inaccuracy) and corrected using the nearest meteorological station. More information on this process can be found in Gangwisch et al.^57^. A 100-m buffer around the single measurement point was considered to assess the relative density of tree cover using the Geographic Information Systems (GIS) QGIS and data were grouped into four groups according to the time of day. The mean air temperature for each time group was then calculated and sorted for the relative class of tree cover density. Hourly model simulations were executed for the reference days increasing tree cover in classes of 10%, from 0 to 100%, and considering 60% of average imperviousness and developed land cover class (e.g., NLCD LC = 24) to account for vehicle path on street patches. The results were then averaged and grouped over the four time periods and sorted by tree canopy density for comparison with measurements.

While the i-Tree Cool Air model has been validated in two previous studies^19,37^, for this application we compared the model results with locally observed air temperature measurements. To overcome discrepancies between the models’ relatively coarse tree cover inputs and hourly air temperature outputs, and the very detailed information gathered during the measurement campaigns, it was necessary to standardize the tree cover data series. To achieve this, we divided the diurnal cycle in four periods of 6-h duration and linearly interpolated the measured and modeled temperature values within each period against tree cover fraction. This resulted in 8 highly significant (*p* < 0.001) and correlated linear regressions (see S5). From these linear regressions, we obtained 8 series of 101 values corresponding to tree cover density from 0 to 100%. Error values were calculated as the absolute deviation from the starting values. A visual illustration of the relation between simulated and measured air temperatures as corrected by tree cover density can also be found in the supplementary information (see S6).

### Scenario analysis for testing hypotheses

To explore hypothesis 1, that dry conditions will decrease the heat mitigation effect provided by trees during very warm periods but improve flood protection due to enhanced infiltration, we simulated air temperature and runoff under various weather conditions and soil moisture regimes for the existing tree canopy cover and impervious cover (base case scenario). The simulation used a 5-yr record of historical meteorological data (Fig. 2) to drive the model, identifying extreme conditions and manipulating boundary conditions during those extremes for testing the hypothesis. For more information about the precipitation regime within this period see Fig. S7 in the supplementary information. For extremely dry and hot conditions, we identified the day with the driest soil moisture conditions during the west-central European drought of 2018^58^, which was August 22nd of 2018, with a simulated soil moisture at 0.215 cm^3^/cm^3^, and an air temperature of 36.7 °C. For extreme wet conditions, June 24th of 2021 was selected, when rainfall totaled 17 mm depth within a one-hour duration, exceeding the 500-yr return interval for the region^59^ and occurring within a multi-week period with above-average rainfall. These observed extremes were then simulated using actual and modified initial settings to evaluate the sensitivity of heat and runoff mitigation to changes in soil moisture and precipitation intensity. In simulations for the hot, dry day of August 22nd 2018, the actual dry soil conditions were contrasted with a high soil moisture (0.24 cm^3^/cm^3^). In simulations for the extreme precipitation event of June 24th 2021, the actual prior wet conditions (soil moisture = 0.29 cm^3^/cm^3^) were contrasted with a dry soil condition (soil moisture = 0.22 cm^3^/cm^3^).

To explore hypothesis 2, that increasing tree canopy and pervious cover decreases incidences of extreme heat and stormwater runoff, we simulated the change in ecosystem services with changes between the base case and green infrastructure case. We modified the initialization separately for the 27 districts of Karlsruhe and simulated heat and runoff mitigation impacts under each case. The green infrastructure case used a tree cover of 30% for each neighborhood, which has been widely adopted as a climate change adaptation measure for cities^28,29^, but has not been achieved yet in any of the Karlsruhe districts (Table 1). It is important to note that we only represented the residential area in each district, denoted as LC values 21–24, and excluded land areas denoted as forest or agricultural land cover. The space for additional tree canopy coverage was taken from impervious surfaces within the district that were not occupied with any green infrastructure. In order to account for tree coverage expanding from pervious to impervious surfaces, for each 1% increase in tree canopy cover, only 0.67% of impervious area was converted to pervious surface (i.e., impervious cover was removed), and 0.33% was maintained as impervious surface but covered by tree canopy.

In both the base case and the green infrastructure case, the simulation was carried out using 5 years of historical climate to evaluate the impacts of ecosystem services. In addition to the standard model output, we also calculated the apparent temperature (Tapp, °C), a heat index of thermal discomfort based on hourly air temperature (Tair, °C), and dewpoint temperature (Tdew, °C) as defined b’ de’Donato et al.^60^:

$$Tapp=-2.653+0.994\times Tair+0.0153\times {Tdew}^{2}$$

The mortality risk for a European elderly population (aged 65 years and older) was found to be significantly increased with Tapp above 31 °C^45^, and we accordingly defined extreme temperatures as Tapp > 31 °C.

## Results

### Assessment of air temperature mitigation data

**Fig. 3:**
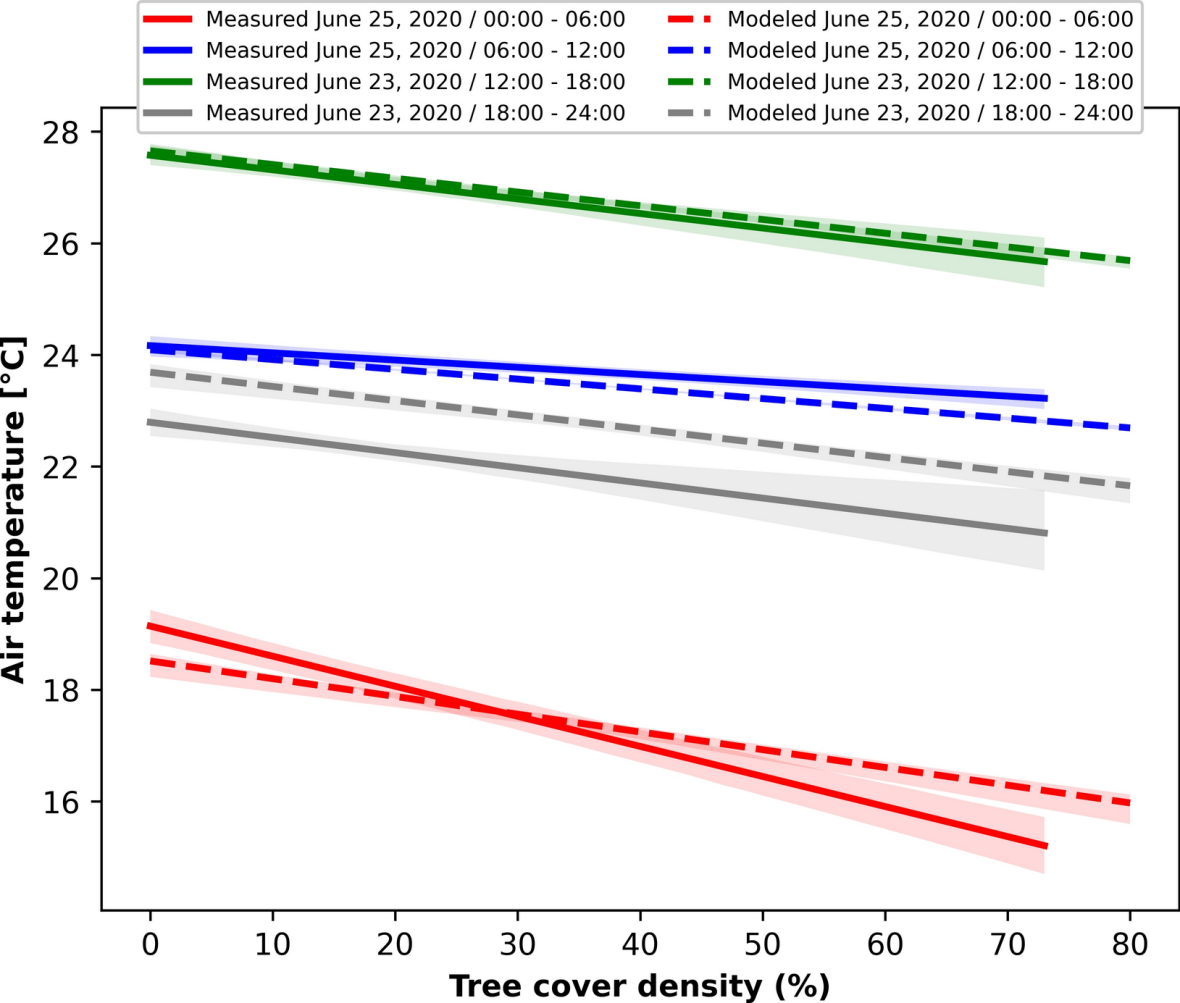
Response of air temperature to tree cover density from mobile measurements performed in Karlsruhe in June 2020 (solid lines) and from modeled results (broken lines) at different times of the day.

The simulated tree cover effect on air temperature was similar to that of the mobile measurements. Increasing the tree cover fraction over urbanized areas can reduce air temperature by up to 5.5 °C in the time interval between 00:00 and 06:00, 1.9 °C between 06:00 and 12:00, 4.0 °C between 12:00 and 18:00, and 4.7 °C between 18:00 and 24:00 (Fig. 3). The correlations for each time slot indicate a statistically significant (*p* < 0.001) proportional effect of tree cover density on temperature reduction. The R-square values range from 0.3 to 0.6 for the measurements and exceed 0.9 in the model regressions. The slope of the regression lines, which express the degree of temperature mitigation per tree cover unit, are lowest between 6 and 12 o’clock (0.013 and 0.017 for measurements and simulations, respectively) and highest between midnight and 6 o’clock (0.054 and 0.027). During nighttime hours, the indirect effect of tree cover, which prevented overheating of the road’s impervious surfaces during the day, leads to reduced longwave radiation emissions and a more pronounced temperature reduction. The larger deviation between simulations and measurements from 18:00 and 24:00, which is depicted graphically in Fig. 3, is due to the high variability of observations (R-square of 0.3). For more on statistical results differentiated by time period see S5 and S6 in the supplementary information.

### Drought impact on air temperature and runoff mitigation by tree cover

**Fig. 4:**
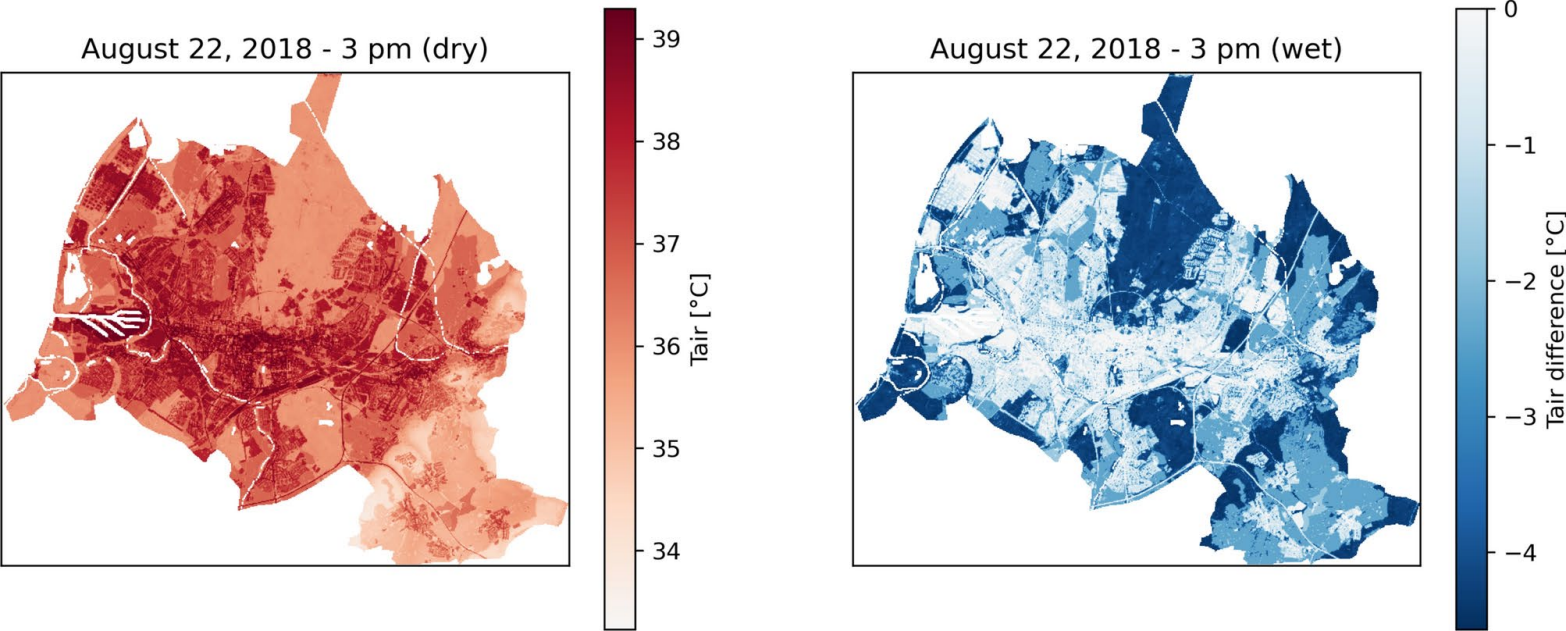
Spatial distribution of modeled air temperature at 3 pm during a very hot day in the historic drought of August 2018. Left Panel: Absolute air temperature values under minimum soil moisture conditions (dry). Right: Air temperature values assuming higher soil moisture conditions (wet). The scale on the right represents the difference in air temperature under wet conditions compared with that under dry conditions. The maps were generated using Python 3.11.4 (https://www.python.org).

The extreme heat mitigation effect of tree cover was tested based on data from August 22, 2018. The day was exceptionally warm and dry, with a daily mean temperature of 25.1 °C and a peak temperature of 36.7 °C at 3:00 PM recorded by the weather station which data were used for driving the model,. Simulations were carried out under both dry and moist soil conditions to test the role of soil moisture on evapotranspiration-based cooling. When simulating the soil to be dry, modelled air temperatures at 3:00 PM varied between 39.3 and 33.2 °C across the whole region (Fig. 4, left), with higher temperatures in the city center and lowest at higher elevations (bottom right of the study area). In the simulations where the soil is simulated to be wet, enhanced evapotranspiration causes up to 4 °C lower temperatures (down to 29.2 °C at 3:00 PM) (Fig. 4, right).

**Fig. 5:**
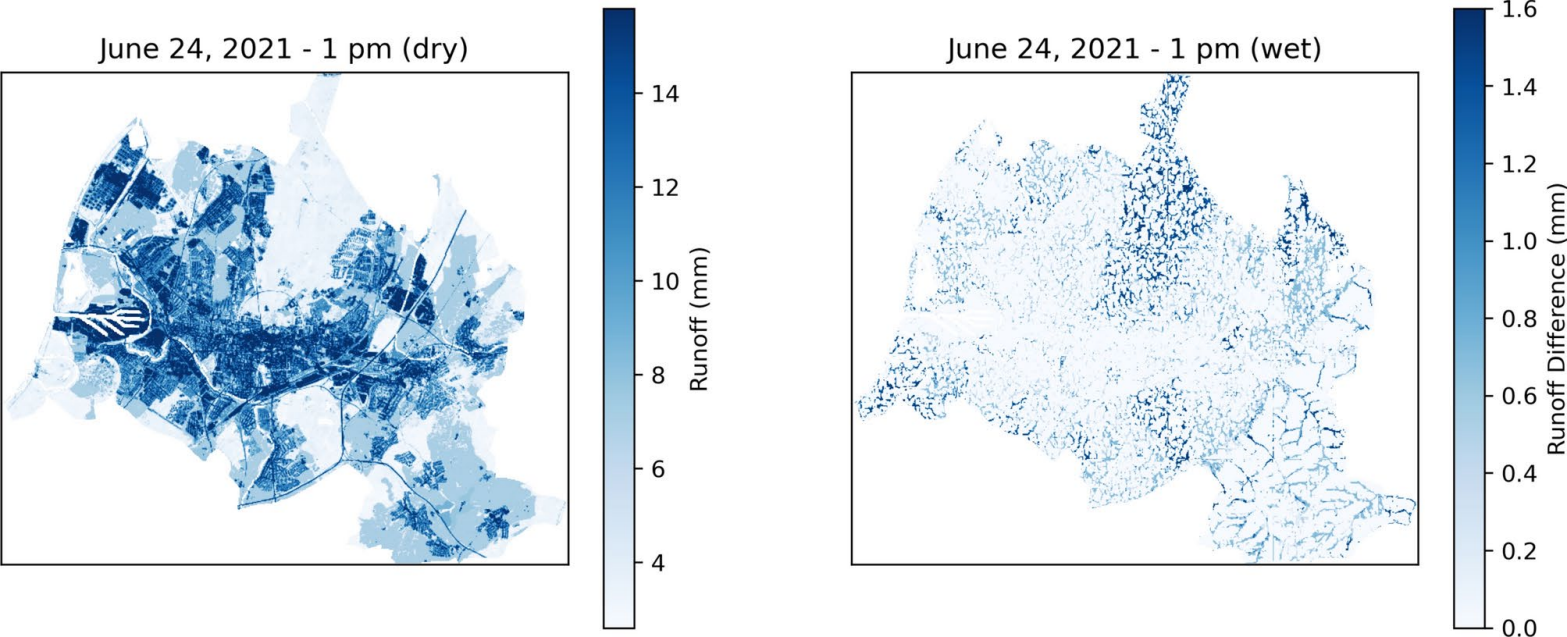
Spatial distribution of modeled surface runoff during a 500-yr return interval heavy rainfall event (17 mm hr^–1^) in June 2021. Left: Surface runoff values assuming drier soil conditions (dry). Right: Surface runoff values under wet soil conditions (wet). The scale on the right represents the difference in surface runoff under wet conditions compared with that under dry conditions. The maps were generated using Python 3.11.4 (https://www.python.org).

The runoff mitigation effect of tree coverage was investigated for the day June 24 in 2021, a day with particularly high precipitation. Simulations were carried out under both dry and moist soil conditions to test the role of soil moisture on stormwater runoff reduction. Runoff varied from 15.8 mm over impermeable surfaces to 2.6 mm for permeable surfaces with litter at the ground (Fig. 5, left). If the soil was simulated to be wet the runoff was higher up to 1.6 mm, with the highest additional outflow in the forested regions (Fig. 5, right). For overall evaluation, it should be noted that 2021 was the year with the highest precipitation within the investigated period, with heavy rainfalls particularly in summer. Therefore, soils can be assumed as wet and runoff was higher compared to drier years such as 2018 or 2020 (see detailed comparison in supplementary Fig. S8).

### Urban greening strategies: scenarios for increasing tree cover

**Fig. 6:**
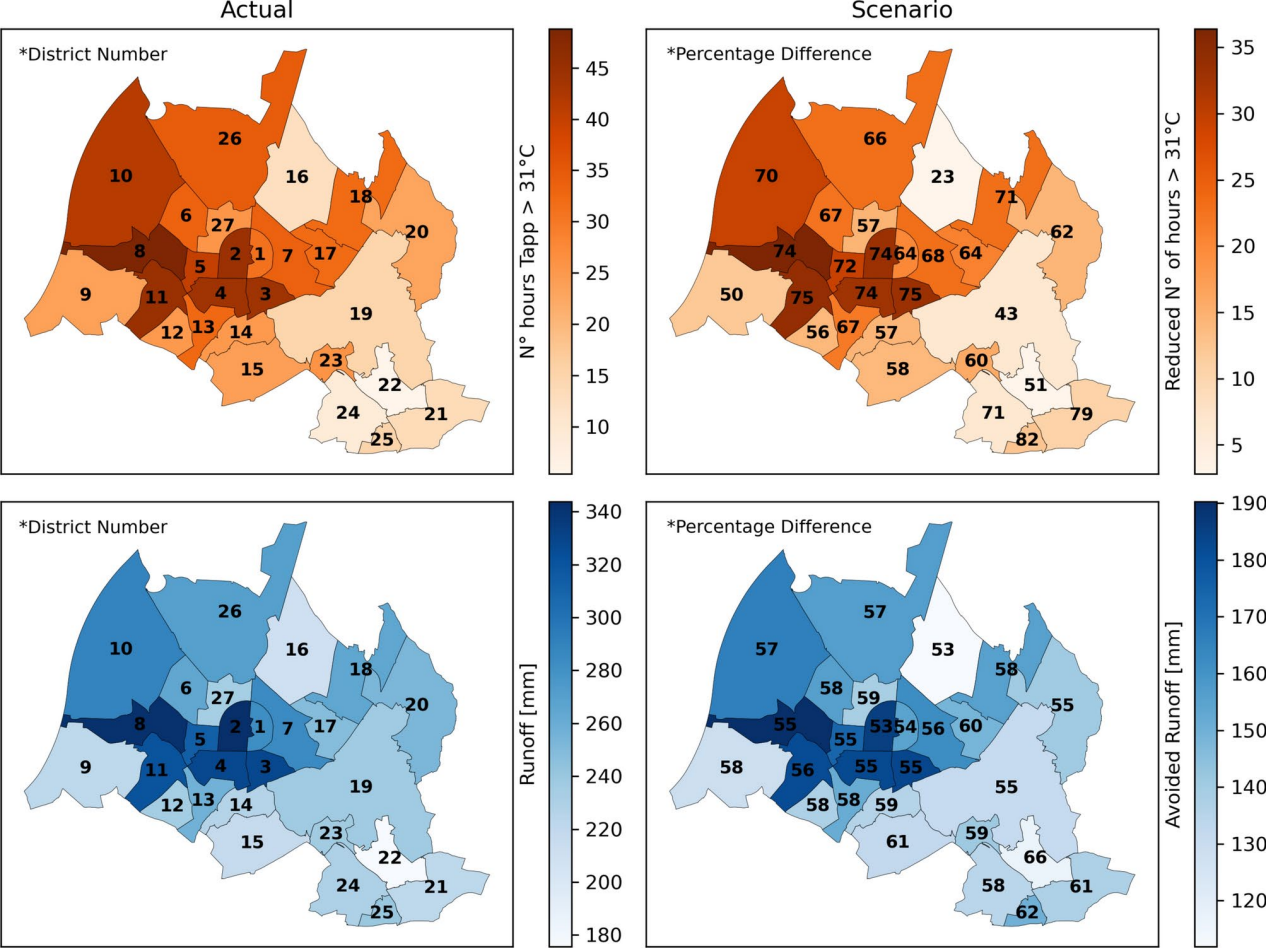
Top: District map showing the average number of hours from 2017 to 2021 with apparent temperature (Tapp) exceeding 31 °C for the base case with actual land cover conditions (Actual) and the green infrastructure scenario with increased tree cover to 30% (Scenario). Bottom: District map showing the average annual stormwater runoff from 2017 to 2021 for the base case with actual land cover conditions (Actual) and the avoided runoff due to the green infrastructure scenario with increased tree cover to 30% (Scenario). Numbers over districts in the left panels indicate district numbers (Table 1), while numbers in the right panels represent the relative percentage difference in improved ecosystem services. The color bars in the left panels indicate the number of hours with Tapp > 31 °C (top) and stormwater runoff (bottom) per each district, whereas the color bars in the right panels show the corresponding reductions in hours and avoided runoff. The simulated new values per district under the scenario are not shown but can be derived as the difference between the left and right panels. The maps were generated using Python 3.11.4 (https://www.python.org).

The effect of a potential urban greening strategy was explored by a comparative analysis of ecosystem service simulations—reduced apparent temperature and avoided runoff—for all districts in Karlsruhe under current tree cover conditions (base case) and under conditions where tree cover was enhanced up to 30% and placed within bioswales (green infrastructure case) (S3). Two essential effects were investigated: mitigation of extreme heat and surface runoff. First, the green infrastructure case generally caused a reduction in the number of hours that crossed a threshold considered to produce significant heat stress for people (Fig. 6, top). In Fig. 6, the number of hours in which the thermal comfort, expressed as apparent temperature (Tapp), crosses a threshold of 31 °C is displayed for current conditions (left). On the right, the number of hours is indicated by which the conditions in each district improve if a tree cover of 30% within bioswales would be established. Our findings indicate that the increased tree cover had a discernible impact in mitigating extreme heat conditions, particularly in the central districts characterized by larger initial impervious areas, where an average reduction of 19.4 h per year (a 64.5% improvement) was simulated. The district with the lowest tree cover (1.6%) over developed land cover classes (Table 1), which is Palmbach (District N° 25), exhibits the most significant relative percentage difference of 82.7%. The lowest percentage difference is recorded in the Waldstadt (District N° 16) which already has the highest tree cover (17.7%) over developed land cover classes (Table 1).

The implications for average runoff conditions of a green infrastructure type tree cover change, which also involves more permeable surfaces, are displayed in Fig. 6 (bottom). Overall, the mitigation pattern is similar to that of extreme heat mitigation, with greater benefits for central districts in Karlsruhe, where up to 151.3 mm of annual surface runoff reduction was simulated (an improvement of 58%). The district with the highest difference in runoff (66.2%) is Hohenwettersbach (District N° 22), which already has the lowest impervious cover (27.8%) among developed land cover classes (Table 1). In contrast, the districts with the lowest differences are Waldstadt (District N° 16), which is characterized by the highest tree cover, and Innenstadt-West (District N° 2), which exhibits the highest impervious cover (73.8%) (Table 1).

## Discussion

While the heat mitigating impact of urban trees has been explored in many case studies^61,62^, only a few studies have investigated temperature impacts of green infrastructure on the city scale, using either statistical^63^ or mechanistic model approaches^64,65^. This includes studies carried out with the i-Tree Cool Air model for Italian cities, showing a clear spatial relation between temperature and green areas that depend on seasonal impacts^37,38^. Similarly, city-scale simulations of stormwater runoff are increasingly available for many cities, which are experiencing and expecting more extreme precipitation^66^. They generally find that stormwater mitigation potential is enhanced by an increased tree coverage and/or perviousness of the ground^16,67^. Urban tree cover designed for stormwater mitigation is a common urban greening target that has been addressed also with the i-Tree Hydro software already^41,42,68,69^. It has been shown that its capacity to reduce runoff is influenced by factors such as tree traits, position, site conditions, and management strategies^70^. The effectiveness of such tree cover diminishes during prolonged and intense rainfall events, as interception is primarily influenced by the amount and frequency of raindrops, reducing the ratio of intercepted to total rainfall^71^. Precipitation thresholds exist where canopy surfaces and soils become saturated, reducing their ability to intercept rainfall or facilitate infiltration. For example, a study in the Shenyang urban area found that as rainfall intensity increases, green infrastructure’s runoff storage capacity declines while demand grows, with only green spaces maintaining a positive balance under 78 mm events (50-year return period), while other urban zones faced deficits^72^. A combined analysis integrating sustainable urban drainage, such as presented here, is a logical step forward, given the efficiency of mitigating extreme runoff and heat depends on filling and draining soil moisture within a mixed landscape of impervious and tree cover, as well as climate boundary conditions.

The current study corroborates that extreme heat and runoff mitigation increase with the deployment of green infrastructure that infiltrates stormwater into soils. First because of evaporation and shading^73^, second because of stormwater storage and increased soil permeability^33^. It, therefore, also confirms hypothesis 1, showing dry conditions decrease the heat mitigation effect provided by trees during very warm periods but improve runoff mitigation due to enhanced infiltration. In contrast, stormwater runoff mitigation is improved under dry initial conditions because the additional water storage to retain incoming precipitation is larger than if the soil (and canopy or surface depression) is already wet. However, the model application indicates that this effect may be of only minor importance, given that saturated conditions are rare across space and time, and unsaturated soils tend to drain at rates greater than the rate at which precipitation arrives. It is important to note that the assessment focused on a high-intensity precipitation event, where the amount of interception of tree cover is relatively low. In contrast, this fraction of rainfall interception is much larger during lower-intensity storm events as demonstrated by Wang et al.^19^ in their application of the i-Tree Hydro model to the urban Dead Run catchment in Baltimore, Maryland. Overall, the model’s capability to represent avoided runoff had been evaluated before over a large range of precipitation intensities during a 5-month leaf-on period in the Great Lakes Urban sewershed. Here, a strong agreement with field measurements was achieved, with 64 L/m^2^ of canopy simulated which was only 4% lower than the measurement-based estimate of 66  L/m^2^^33^.

The model simulations for Karlsruhe failed to reject hypothesis 2, that increasing tree canopy and pervious cover decreases incidences of extreme heat and stormwater runoff. Particularly, the number of hours that are hot enough to do damage to human health could be substantially reduced by a realistic afforestation scenario that integrates stormwater capture. The impact gets even larger, if it is considered that the most vulnerable people in Karlsruhe are concentrated in the districts that are most exposed to extreme heat events. The results, therefore, could also be used for estimating lives saved with a specific greening scenario, as has been done by combining i-Tree Cool Air model cooling estimates with epidemiological functions for US cities^35,36^. Earlier model assessments of air and dew point temperature were evaluated with urban weather stations^34^, showing a good agreement for defined periods even under relatively dry conditions^37^. However, the number of stations in these studies were relatively few and were fixed at places with defined boundary conditions (e.g. distance to vegetation, pervious coverage). The mobile data used in this investigation have the advantage of covering a large spatial range of tree cover densities within the city where the investigation actually took place^57^. This comes with the disadvantage of a campaign-based evaluation being reduced to single points in time.

The methodology presented here can be used as a decision support to prioritize areas within a city to implement green infrastructure. Without consideration of demographic data, the city districts achieving the greatest potential reduction in extreme heat and stormwater runoff are located in the central north-western part of Karlsruhe (Fig. 6). This district pattern of greatest benefits changes when considering the relative distribution of vulnerable people, defined simply by age (> = 65 years). Due to a higher share of older, more vulnerable people at the periphery of the city, additional green cover in these districts was more effective (weighted distribution shown in supplementary Fig. S9). Integrating the distribution of vulnerable populations allows for more targeted green infrastructure, maximizing social impact. This analysis highlights the need to consider not only land cover conditions, such as impervious surface and tree cover, but also factors that represent the vulnerability of the people living in the respective area. Relying solely on land cover and age may still overlook other critical social and economic factors, such as income or access to resources, which can affect how populations experience extreme heat or stormwater runoff^74,75^.

We acknowledge that the recommendation to concentrate green infrastructure in the developed areas of the city may produce conflicts with the current building structure. However, a comprehensive concept focusing on connectivity, accessibility and quality of the green spaces^76^, also considering benefits in air quality or biodiversity^77,78^, can be applied for political decision making and discussion with stakeholders. Also, many places at or close to *s*treets, sidewalks, and other paved areas that can be used for afforestation within bioswales can still be found. In particular, parking spaces might be suitable targets since, on the one hand, cities are more and more transformed to prefer other transport options than individual cars^79^, and on the other hand, parking spaces are often in need for shading opportunities that justify a partial transformation without abandoning the function itself^80^.

As also shown by our study, mitigation of extreme heat events depends considerably on water availability, potentially requiring irrigation in periods of prolonged drought. Reduced soil water availability significantly limits transpiration by restricting stomatal conductance and disrupting water relations^81^, processes that directly contribute to temperature regulation in urban and natural environments^82,83^. Increasingly dry conditions driven by climate change further exacerbate this limitation, significantly reducing vegetation’s cooling capacity^39^. Without sufficient soil water, vegetation struggles to perform its natural cooling functions, reducing its capacity to regulate temperatures^23,84^. This underscores the importance of managing soil water availability, including the potential need for irrigation during prolonged drought periods, to ensure that green spaces can continue to provide effective heat mitigation^85^. Neglecting this requirement will lead to damages to trees and other plants due to extreme heat and drought which then in turn prevent the provision of ecosystem services even after the stressful conditions have ended^86,87^. Although the city of Karlsruhe may not currently be in direct danger of suffering water shortages, the medium-term climate scenarios indicate drought will reduce groundwater availability in Germany^88^. This suggests that sustainable urban drainage design that removes impervious cover as well as builds additional reservoirs to store water runoff is an increasingly urgent issue to be addressed by cities^89^. Given that these stormwater measures would also be beneficial to reduce extreme heat damages, this supports the need to synergistically look into water as well as energy management of a city since they are interlinked. Also, this study shows that not considering both together might underestimate the overall impact considerably.

While we emphasize the importance of mitigating extreme heat and stormwater impacts, other factors can equally contribute to human health or infrastructure vulnerability. For example, air quality is worsening under extreme heat conditions which can contribute to pulmonary diseases^90^. This is partly due to the emission of organic compounds from plants that participate in ozone formation and are particularly high at heatwave events^91^. We would also like to point out that the current study neglects species-specific plant properties. It has been shown that plant traits such as leaf area density, canopy conductance, crown structure or foliage longevity, influence ecosystem service efficiency^20,70,92,93^. Thus, considering such tree functional traits would improve the quantification of ecosystem services and support decisions of tree species selection. Considering species-specific properties and also including deposition and emission of chemical compounds would also enable to consider further trade-offs. For example, while dense canopies benefit shading and pollution removal, they may also increase water use or emissions of reactive compounds, exacerbating issues like tropospheric ozone and particle formation under future climatic conditions^94^. Additionally, the lack of explicit spatial distribution of green space within a grid should be addressed to better optimize the balance between the supply and demand for ecosystem services^95,96^, thereby supporting urban planning and management strategies^97,98^. Furthermore, the analysis does not consider potential negative ecosystem services, such as tree management challenges^99^ or pollen allergenicity^100^, which could partially offset the benefits and highlight the trade-offs and limitations of urban greening strategies^101^.

This study highlights the dual benefits of reducing stormwater runoff and mitigating extreme heat through green infrastructure. However, these interventions also necessitate navigating synergies and trade-offs across environmental, social, and economic dimensions. For example, prioritizing areas based on environmental potential (e.g., heat mitigation, flood risk) versus social needs (e.g., vulnerable populations) illustrates a key synergy between urban climate resilience and social equity^102^. Additionally, water availability represents a critical axis of synergy and trade-off. While vegetation in green infrastructure systems enhances heat mitigation via evapotranspiration, it also increases water demand during prolonged droughts. This underscores the need for sustainable urban drainage systems that not only manage runoff but also incorporate storage reservoirs to buffer against water scarcity, particularly as climate-driven trade-offs between water retention and cooling benefits vary by region, with arid environments favoring retention and energy-limited climates favoring cooling^21^. Finally, the modeling approach employed in this study is designed primarily as a tool for initial city planning, providing broad-scale guidance through the use of readily available data and enabling relatively rapid simulations across multiple scenarios. High-resolution analyses of the urban microclimate, which account for modifications to the urban boundary layer by buildings and excess heat, can complement this approach by supporting more detailed planning at finer scales, such as within specific street canyons^103^. Additionally, comparing model assessments of surface runoff with Karlsruhe flood risk maps^104^ could improve the identification of areas where green infrastructure mitigates runoff and complements flood management strategies.

## Conclusions

This study has demonstrated that extreme heat and runoff mitigation significantly improve for cities with an increase in green infrastructure that integrates tree cover and sustainable urban drainage. This is particularly relevant given climate change and predictions of extreme temperature and precipitation in many cities. Furthermore, it has been shown that the efficiency of extreme heat mitigation is closely tied to water availability, which represents an essential prerequisite for evapotranspiration processes. On the other hand, stormwater runoff mitigation is (slightly) enhanced under dry initial conditions. Additionally, our study has confirmed that green infrastructure strategies can be strategically designed starting from the analysis of land use, tree cover and impervious cover in city districts to reduce extreme weather events harmful for human health. An integrated approach to prioritize city districts for green infrastructure projects that enhance ecosystem services will also consider where the population is most vulnerable and will benefit most. The study also shows that synergies or trade-offs of management options regarding urban greening can be assessed using sophisticated decision support tools such as a water and energy model able to consider various scenarios.

## Supplementary Information

Supplementary Information.

## Data Availability

The sources for geographical data, weather data, and population statistics were indicated in the 'Model inputs of map data and weather’ section of the Methodology. The datasets used and/or analyzed during the current study are available from the corresponding author upon reasonable request.
