# Supplementary material for: Mitigation potential of urban greening during heatwaves and stormwater events: a modeling study for Karlsruhe, Germany
Source: Sci Rep. 2025 Feb 13;15:5308. doi: 10.1038/s41598-025-89842-z (PMC11822192; doi:10.1038/s41598-025-89842-z)
Supplement: Supplementary file 1 — Supplementary Information. [file 41598_2025_89842_MOESM1_ESM.docx]

**SUPPLEMENT**


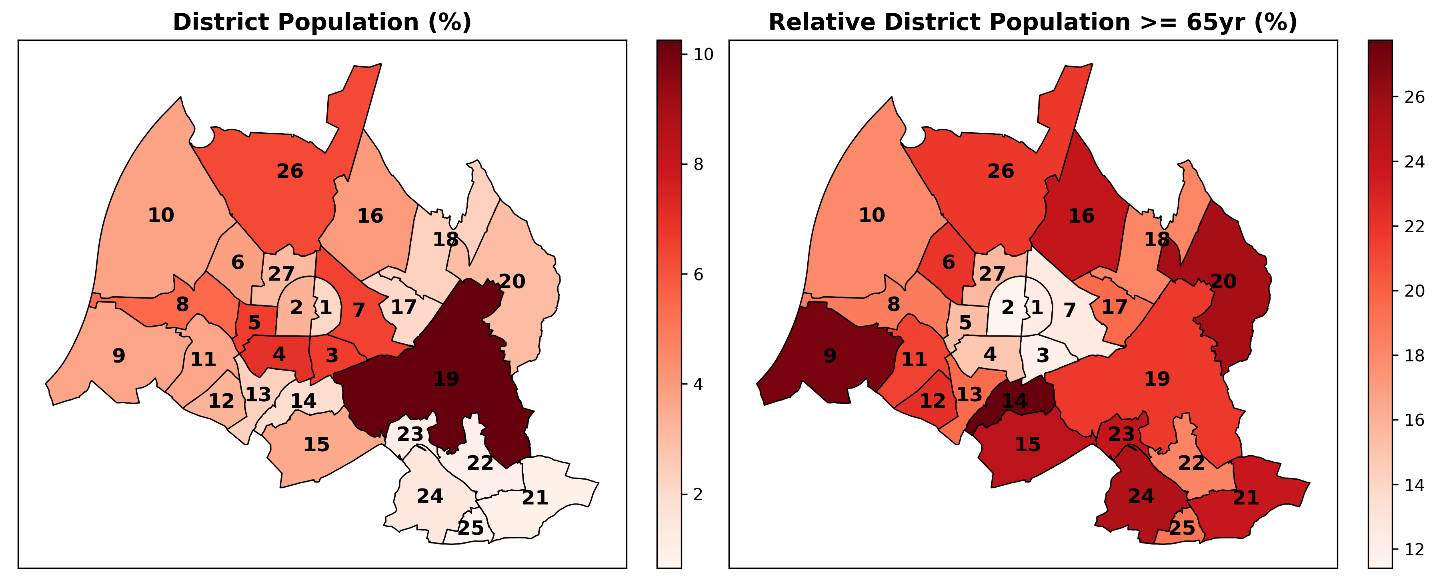


**S1.** Left: Percentage distribution of Karlsruhe's population across districts. Right: Relative percentage of population aged 65 or older for each district of Karlsruhe. The maps were generated using Python 3.11.4 (https://www.python.org).

**S2.** Land cover statistics for the city of Karlsruhe per each class for relative area, mean Tree Cover (TC), mean Impervious Cover (IC) and Population density.

| **Land use**  **class** | | **Area (%)** | **Mean**  **TC (%)** | **Mean IC**  **(%)** | **Population**  **(%)** |
| --- | --- | --- | --- | --- | --- |
| 11 | Open Water | 3.1 | 1.0 | 0.3 | 0.0 |
| 21 | Developed, Open Space | 5.6 | 0.7 | 1.1 | 1.2 |
| 22 | Developed, Low Intensity | 3.6 | 0.3 | 1.3 | 12.0 |
| 23 | Developed, Medium Intensity | 20.5 | 0.9 | 11.8 | 44.6 |
| 24 | Developed High Intensity | 13.1 | 0.3 | 9.5 | 40.4 |
| 31 | Barren Land (Rock/Sand/Clay) | 0.7 | 0.1 | 0.1 | 0.0 |
| 43 | Mixed Forest | 32.7 | 14.4 | 3.1 | 0.0 |
| 52 | Shrub/Scrub | 0.1 | 0.0 | 0.0 | 0.0 |
| 71 | Grassland/Herbaceous | 1.5 | 0.2 | 0.1 | 0.1 |
| 82 | Cultivated Crops | 18.9 | 1.8 | 0.7 | 1.7 |


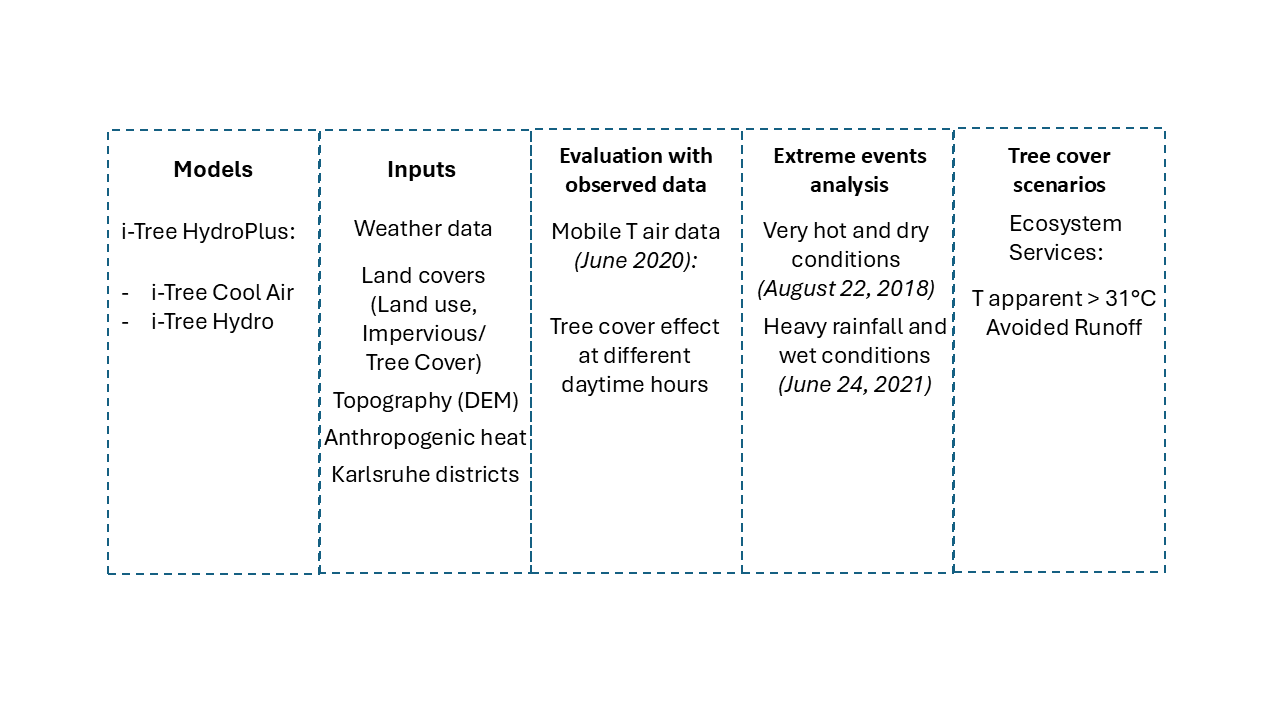


**S3.** Workflow of the adopted methodology, including coupled hydro-climatology models for water and energy balances (Models), processing of land cover and meteorological inputs (Inputs), validation with observed air temperature data (Evaluation with observed data), analysis of extreme events (Extreme events analysis), and assessment of ecosystem services under tree cover scenarios (Tree cover scenarios).


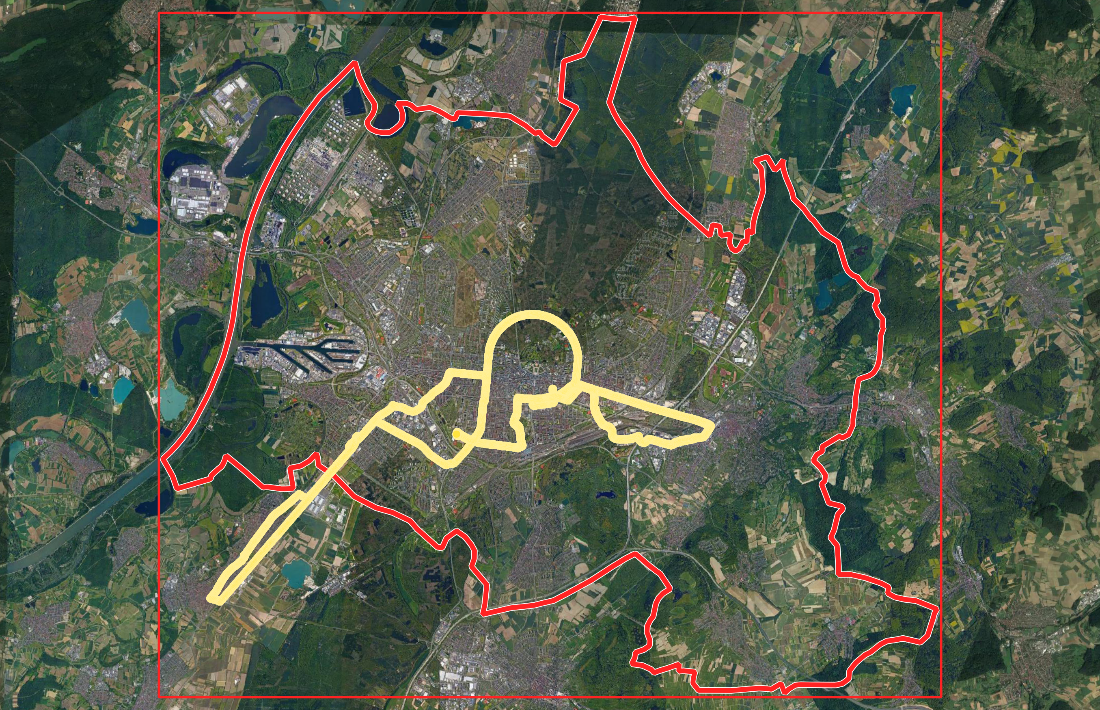


**S4.** Route taken for mobile temperature measurements (yellow) through the city of Karlsruhe (borders in red). The map was generated using QGIS 3.32.2 (https://www.qgis.org/).

**S5.** Statistical results of linear interpolations of measurements and model values (p < 0.001) per each time period with respect to tree cover density in 8 linear regressions. The column VAR indicates with V measurements and M model results, followed by the time interval. DELTA is the SLOPE absolute value multiplied by 100 and SENS is the ratio between model and measure SLOPE.

| ***VAR*** | ***R2*** | ***SLOPE*** | ***INTERCEPT*** | ***DELTA*** | ***SENS*** |
| --- | --- | --- | --- | --- | --- |
| **V0006** | 0.634 | -0.054 | 19.140 | 5.4 | 0.50 |
| **M0006** | 0.953 | -0.027 | 18.391 | 2.7 |  |
| **V0612** | 0.400 | -0.013 | 24.165 | 1.3 | 1.31 |
| **M0612** | 0.995 | -0.017 | 24.065 | 1.7 |  |
| **V1218** | 0.518 | -0.026 | 27.576 | 2.6 | 0.85 |
| **M1218** | 0.982 | -0.022 | 27.596 | 2.2 |  |
| **V1823** | 0.327 | -0.027 | 22.792 | 2.7 | 0.81 |
| **M1823** | 0.947 | -0.022 | 23.589 | 2.2 |  |


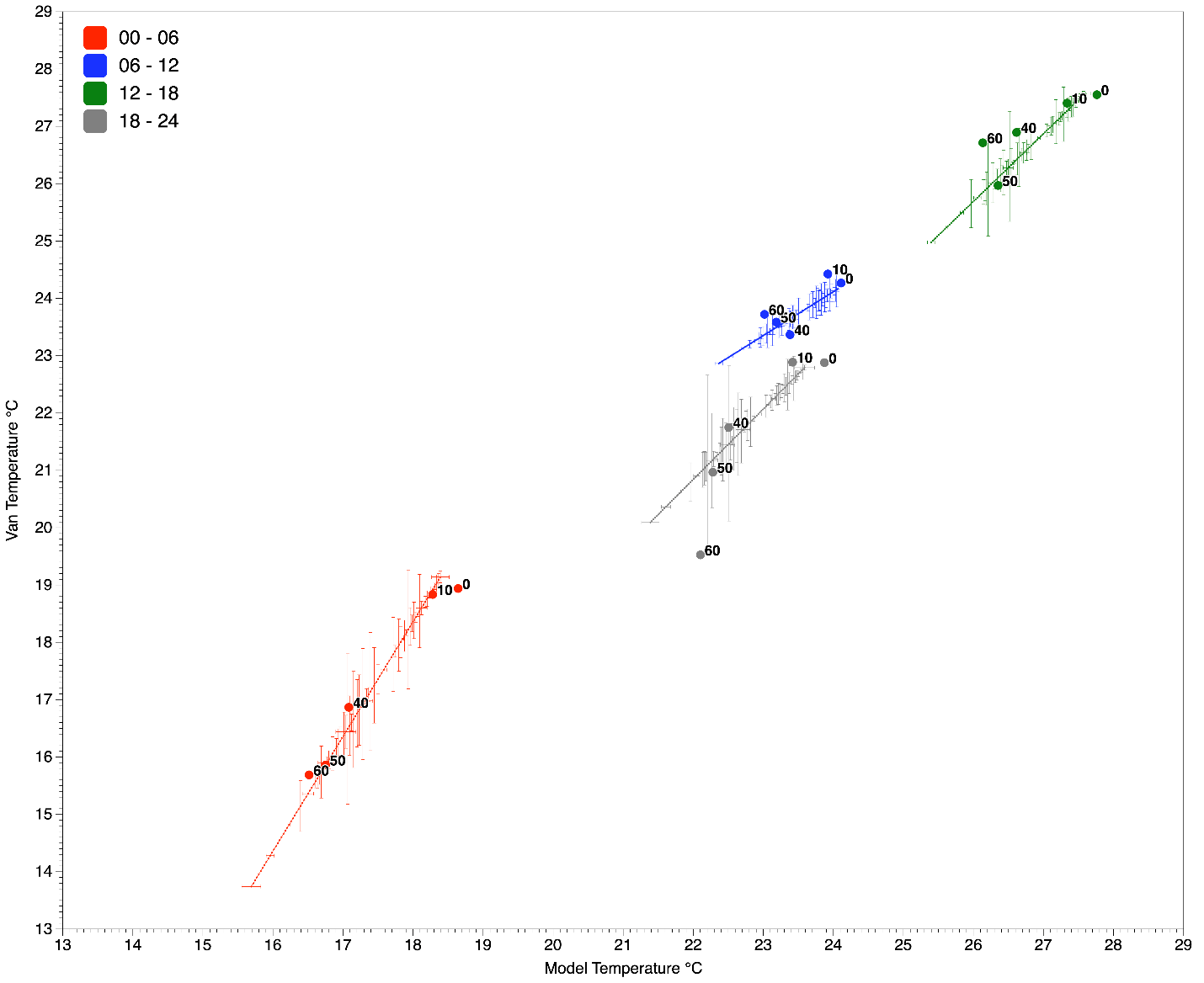


**S6.** Linear regressions between simulated and measured temperature values for each of four periods during the day. Each regression ranges from tree cover 0, in the upper right, to tree cover 100, in the lower left. For illustration, tree cover density values are indicated for each regression line at common temperature correlations. Horizontal error bars reflect variations in the simulated temperatures while vertical lines indicate the variability of the measurements.


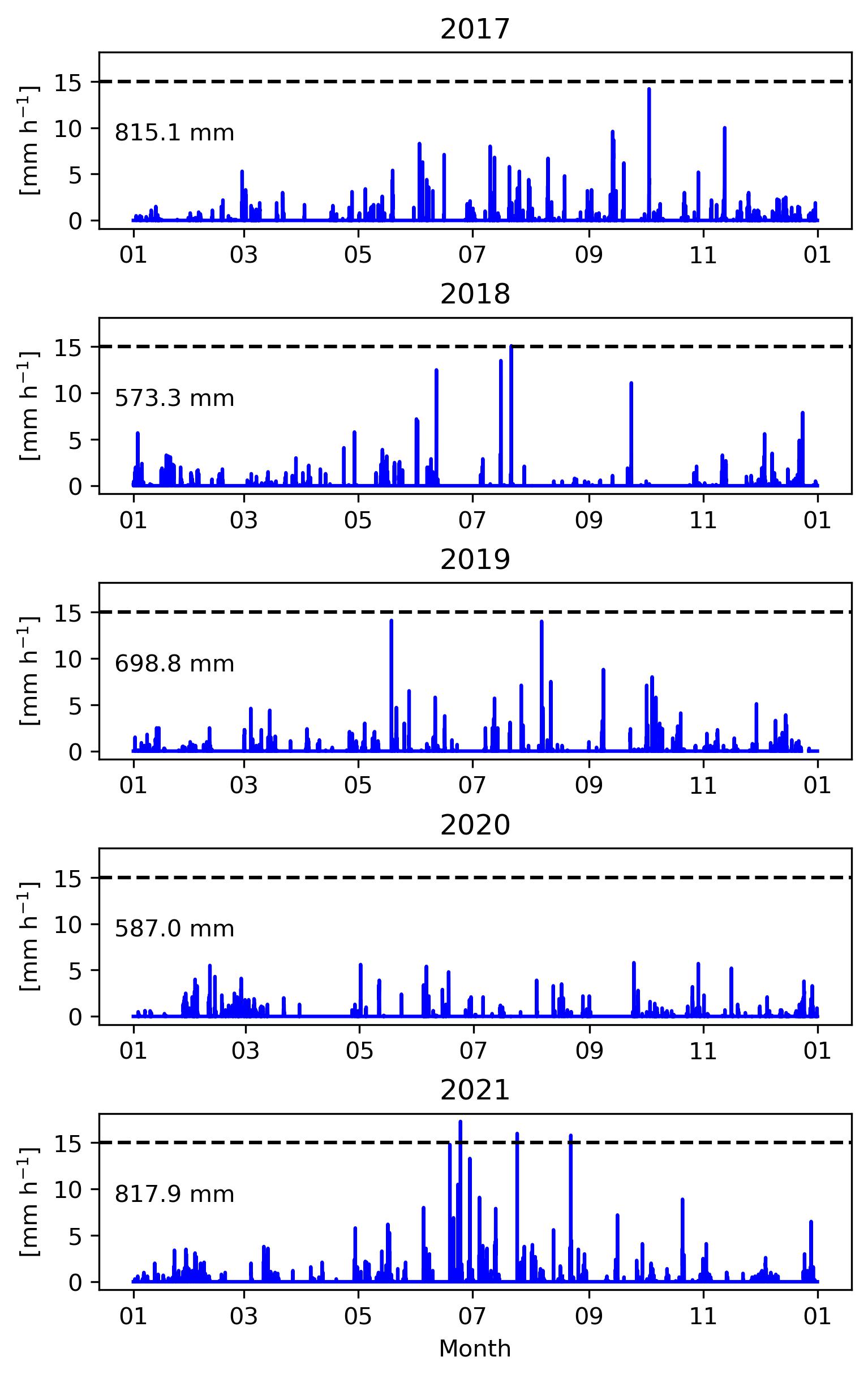


**S7.** Hourly precipitation in Karlsruhe from 2017 to 2021 with the 15 mm threshold for heavy rainfall events (indicated as dashed line).


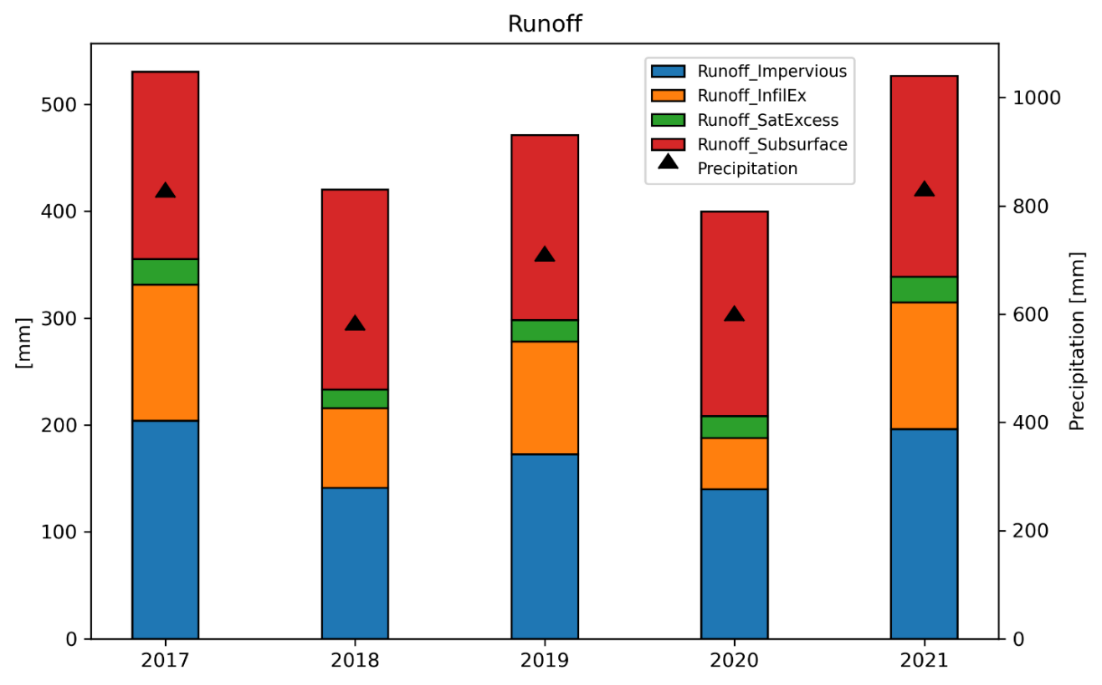


**S8.** Budget of runoff components (impervious, infiltration excess, saturation excess, subsurface) from 2017 to 2021 compared with annual precipitation.


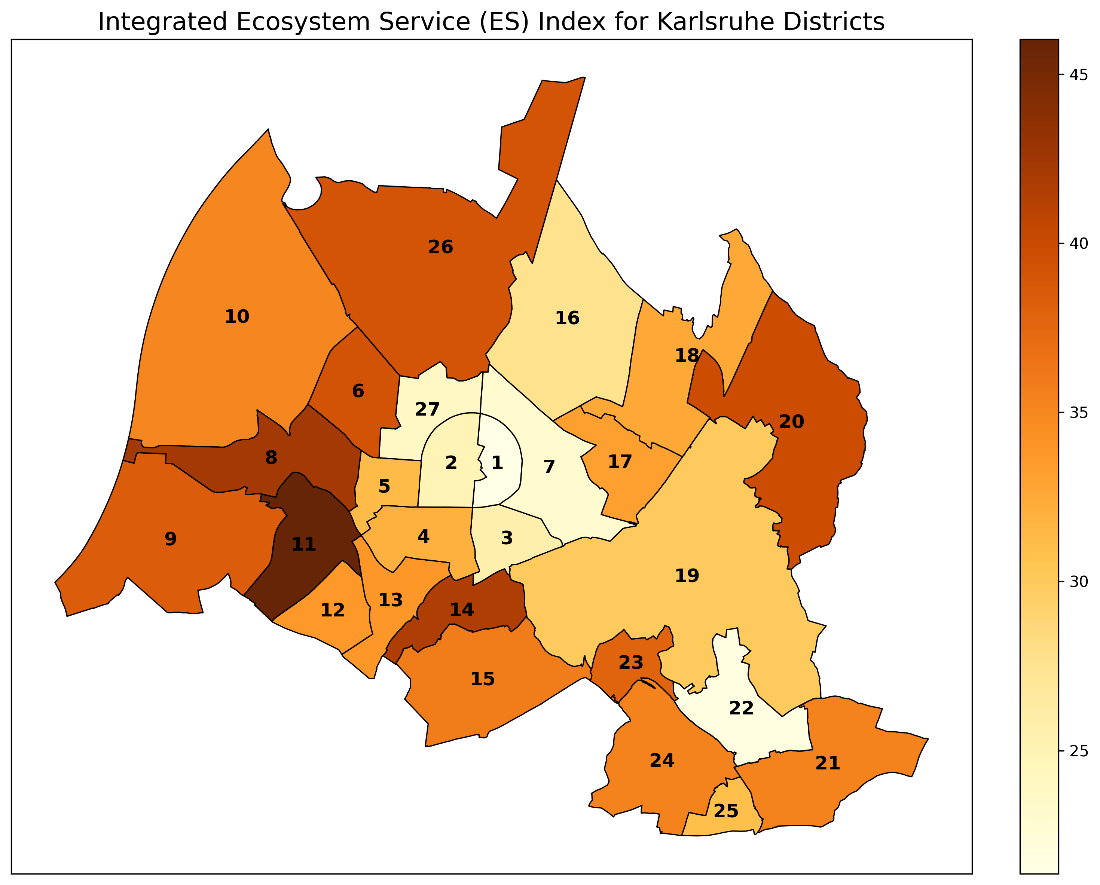


**S9.** Ecosystem Service (ES) Index that sums the number of avoided days with temperatures exceeding 31°C and the average avoided annual runoff per each district (Fig. 6), with values normalized by the ratio of the population aged 65 or older over the total population in each district of Karlsruhe (Fig. S1). The ES index identifies districts where the proposed green infrastructure scenario can provide the greatest benefits in mitigating environmental stresses, while also prioritizing high-exposure areas among vulnerable populations. The map was generated using Python 3.11.4 (https://www.python.org).
